# Long-term results of dorsal neuroma/nerve transposition in the surgical management of Morton’s neuroma and correlation with intraoperative anatomical variations

**DOI:** 10.1186/s13018-022-02910-2

**Published:** 2022-01-15

**Authors:** Manjunath Koti, Nicola Maffulli, Muwaffak Al-Shoaibi, Michael Hughes, Jack McAllister

**Affiliations:** 1 Princess Royal University Hospital, Kings College Hospital NHS Trust, Orpington, Kent, BR6 8ND UK; 2 Barts and the London School of Medicine and Dentistry, Centre for Sports and Exercise Medicine, Mile End Hospital, Queen Mary University of London, 275 Bancroft Road, London, E1 4DG UK; 3 Homerton University Hospital, London, E96SR UK; 4 Chelsfield Park Hospital, Bucks Cross Road, Chelsfield, Orpington, Kent, BR6 7RG UK

**Keywords:** Morton’s neuroma (MN), Dorsal nerve transposition (DNT), Dorsal nerve relocation, Dorsal neurectomy, Dorsal neurolysis, Deep transverse intermetatarsal ligament (DTIML)

## Abstract

**Background:**

Morton's neuroma (MN) is a common cause of forefoot pain. After failure of conservative management, surgical procedures include neurectomy or neuroma preserving procedures; resection of deep transverse intermetatarsal ligament only (DTIML), dorsal neurolysis, dorsal nerve transposition (DNT).

**Objectives:**

This retrospective study evaluates the long-term results of open DNT, and it also reports anatomical variants in the plantar interdigital nerve.

**Material and methods:**

The study included 39 patients (30 females and 9 males) who were treated for MN between 2002 and 2016.

**Results:**

The mean pre-operative Giannini score of 13 (0–30) improved to 61 (20–80) (*p* < .0001), with only 6 patients scoring less than 50 (poor). Using Coughlin’s criterion for overall satisfaction, 9 patients (23%) reported excellent, 18 patients (46%) good, 6 patients (15%) fair and 6 patients (15%) reported poor results. In the long term, 25 patients (64%) had no pain, 8 patients (20%) had mild pain, and 6 patients (16%) had severe pain. Ten patients (26%) reported normal sensitivity in their toes, 26 patients (66%) had numbness, and 3 patients (8%) reported dysesthesia in their toes. Twenty-two patients (56%) could wear fashionable shoes, 11 patients (28%) comfortable shoes, and 6 patients (16%) modified shoes. Regarding walking distance, 30 patients (77%) had no limitation, and 9 patients (23%) reported some limitation. Nineteen per cent regretted having surgery. Around 40% (17 out of 43 web spaces) showed anatomical variations in either the nerve or in the web space and we could not identify any specific risk factors in relation to the outcome.

**Conclusion:**

Dividing the DTIML or dorsal neurolysis should be considered as the primary surgical treatment and, if this fails, neurectomy would be an option. DNT can be considered if one is concerned about stump neuroma, but this may be technically demanding and in some patients it may not be possible.

*Level of Evidence*: Level IV - Case Control Retrospective study.

## Introduction

Morton’s neuroma is probably the most common nerve lesion in the lower limb. Of multifactorial aetiology, its main cause is probably chronic repetitive trauma of the interdigital nerve against the deep transverse intermetatarsal ligament (DTIML).

Excessive weightbearing stress on the forefoot, caused by wearing pointed and high-heeled shoes causing hyper dorsiflexion of the metatarsophalangeal (MTP) joint, can produce microdamage by stretching of the interdigital nerve against the DTIML as it courses dorsally from the plantar aspect, close to the distal aspect of the intermetatarsal bursa [1]. Morton’s neuroma is a combination of perineural fibrosis, neural oedema and demyelination. As the scar tissue around the nerve accumulates, patients experience intermittent burning, paraesthesia and loss of sensation in the toes.

When conservative measures fail, the neuroma is often treated by injections, decompression (open or minimally invasive) or excision through dorsal or plantar approaches.

Gilmour in 1973 described a neuroma preserving procedure [2]. After dividing the deep transverse intermetatarsal ligament, through a dorsal approach, the neuroma is freed from the surrounding tight soft tissues. Proponents of this approach suggest that relieving the mechanical stresses on the nerve by dividing the DTIL is the key aspect for successful surgical treatment and, by preserving the neuroma, some or normal nerve function can return [3–5]. Furthermore, by not resecting the neuroma, the risk of developing a true stump neuroma is avoided, preventing a major the problem of neurectomy [6, 7]. The aim of this study was to report the long-term results of dorsal neuroma/nerve transposition by a single surgeon and describe how the anatomical variants of the nerve may impact the final outcome of the procedure.

## Material and methods

### Data collection

**Fig. 1:**
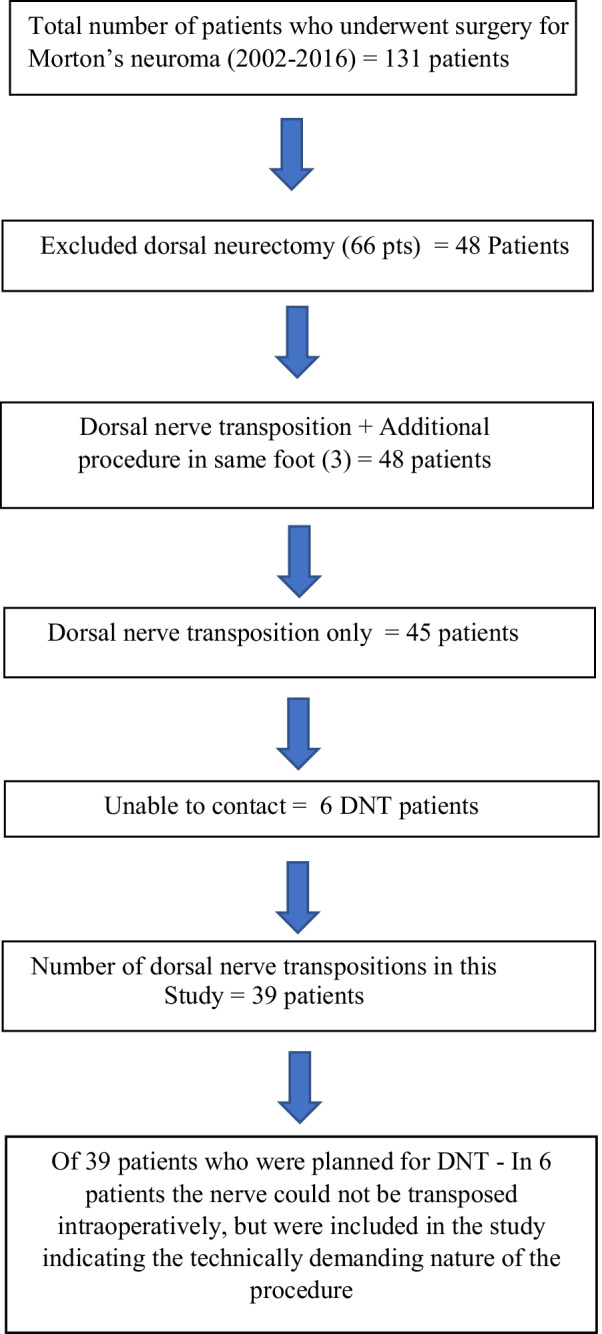
Flow chart of the total number of patients included after exclusion criteria

With the data obtained from the hospital database (Hospital Coding Department and Business Intelligence Unit), we identified 48 patients who had undergone Dorsal Neuroma Transposition between 2002 and 2016. Six patients could not be traced and 3 patients who had additional procedures (bunionectomy in one, PIP joint fusion in another and a sliding metatarsal osteotomy of the 3rd metatarsal) were not included in this study (Fig. 1).

In total, 39 patients (43 web spaces) underwent dorsal neuroma/nerve transposition including 6 patients where the nerve could not be transposed intraoperatively. All surgical procedures were performed by a single surgeon (JM).

### Outcome measures

The primary outcome measures were the Interdigital neuroma scoring system (Table 1) and Coughlin’s overall patient satisfaction criteria (Table 2).

**Table 1:** Interdigital neuroma clinical evaluation score Giannini et al. [8]

Score parameter	20	10	0
Pain	None	Mild	Severe
Maximum walking distance	Without limitation (greater than 6 blocks)	Some limitation (2–6 blocks)	Severe limitation (less than 2 blocks)
Sensitivity	Normal	Numbness	Dysesthesia
Footwear requirement	Fashionable conventional shoes	Comfortable footwear or shoe insert	Difficulty with any shoe wear

**Table 2:** Coughlin’s criteria of overall patient satisfaction [9]

Excellent	Without problems, very satisfied, mild or no pain, walks without difficulty
Good	A few problems, satisfied with mild pain. Walks without difficulty or with mild difficulty, would still have the surgery
Fair	Moderate pain, some mild difficulty in walking, has reservations about surgery
Poor	Continued pain, little improvement in walking, regrets having surgery

## Results

Thirty-nine patients (43 web spaces) responded to our postal and telephone questionnaires. Of them, 30 were females and 9 were males. The right 2/3 webspace was involved in 4, the right 3/4 in 11, the left 2/3 webspace in 11 and the left 3/4 in 17 patients. The average age of the patients at surgery was 52 (range 31–79). The mean pre-operative Giannini score was 13 (range 0–30), increasing post-operatively to 61 (range 20–80) (*p* < 0.0001).

According to Coughlin’s criteria for overall satisfaction, 9 patients (23%) reported excellent, 18 patients (46%) good, 6 patients (15.4%) fair and 6 patients (15.4%) reported poor results.

DNT produced excellent to fair results in 33 patients (84%) with a score above 50. Six patients (15%) had score of less than 50. At long-term follow-up, two-thirds of the patients had no pain, 20% had mild pain, and 15% had severe pain.

One patient with three neuromas, all dorsally transposed reported a good result. Two patients had bilateral neuromas dorsally transposed; one had a good result on both sides, and the other had an excellent result in one foot and a fair result in the opposite foot.

Overall, Coughlin’s overall patient satisfaction criteria matched with the Giannini scoring system, showing successful results (excellent to fair) in 85% and poor results in 6 (15%) of patients. Sixty-seven per cent of patients would still undergo surgery, 14% had some reservations about surgery, and 19% regret for having surgery.

Ten patients (around a quarter) (26%) had normal sensitivity in their toes, 26 patients (two-thirds) (66%) had numbness, and 3 patients (8%) reported dysesthesia in their toes.

Twenty-two patients (more than half) (56%) could wear fashionable shoes, 11 patients (28%) wore comfortable shoes, and 6 patients (16%) wore modified shoes.

More than three-fourths (77%) had no limitation in walking; 23% of patients had some limitation (2–6 blocks).

### Intraoperative findings and anatomical Morton’s neuroma variations

Of the 39 patients (43 web spaces), 23 (26 web spaces) showed normal anatomy of the interdigital nerve with a neuroma at the “Y” bifurcation and underwent a standard DNT after dividing the deep transverse intermetatarsal ligament and mobilising the nerve. Once transposed dorsally, a vicryl sling was fashioned between the metatarsals acting as a hammock to maintain the dorsal position of the transposed neuroma.

**Fig. 2:**
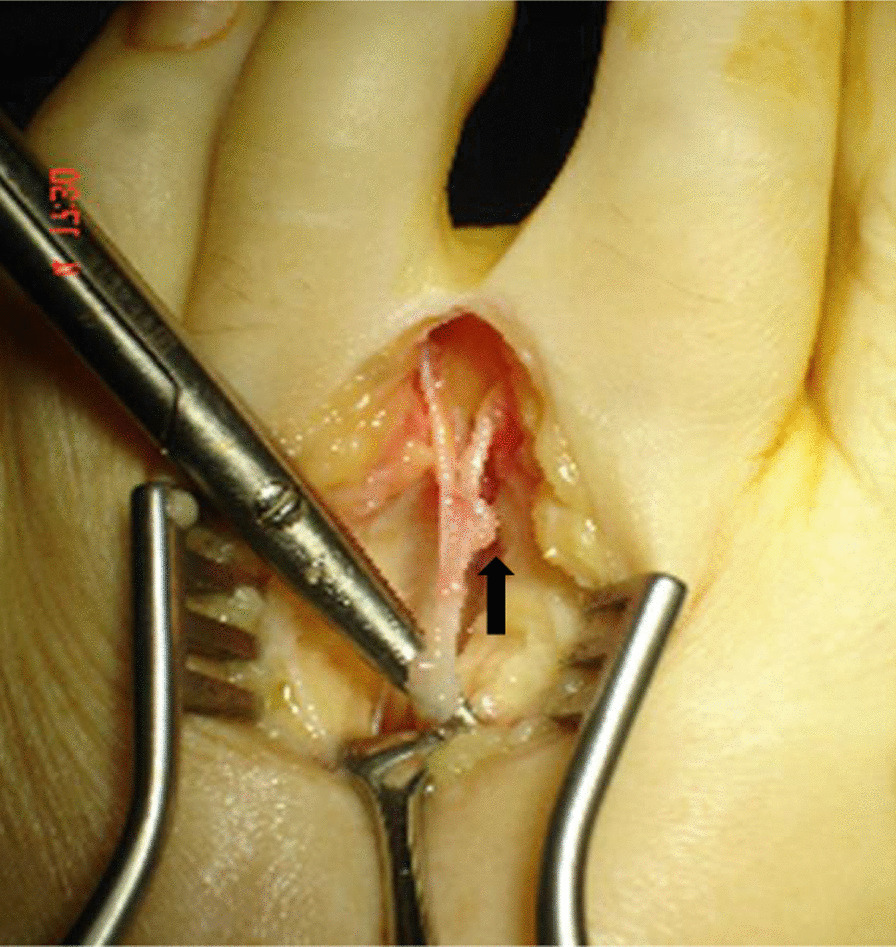
Arrow pointing towards the neuroma mainly on the divisional branch to the 3rd toe

The remaining 16 patients (17 web spaces) (40%) showed some anatomical variation either in the web space or of the interdigital nerve. Two patients had prominent veins in the web space, 1 had an unusual branch from 2/3 web space joining the 3rd interdigital nerve, 2 patients had a main divisional branch to 3rd and a small branch to the 4th toe, 3 patients had a neuroma on the branch to the 3rd toe (Fig. 2), in 2 patients the nerve had an abnormal course passing under the metatarsal head distally, and in 1 patient under the 3rd metatarsal shaft. In 6 patients either the nerve or one of the divisional branches was thickened or tethered, 3 patients had bursal thickening and 3 patients had a prominent plantar branch. In 8 patients, a neuroma was not seen at surgical exploration. In 6 patients, the nerve/neuroma could not be transposed.

Table 3 gives the further details of the intraoperative anatomical variants identified.

**Table 3:** Details of the 17 web spaces with intraoperative anatomical variants

Pt no	Age/sex	Web space	Anatomical variation	Neuroma	Transposed	Outcome
1	61 F	L 2/3	Extensive vascular leash/plexus dorsal to bifurcation	No neuroma seen Nerve normal	Yes, but difficult	Poor
2	68 M	L 3/4	Proximally unusual large branch from adjacent 2/3 web space joining the 3rd interdigital nerve	Neuroma proximal to bifurcation	Not possible	Good
3	58 F	L 3/4	Main division branch to 3rd toe, smaller divisional branch to 4th toe	No neuroma seen Nerve normal	Yes, but difficult	Good
4a	61 F	R 3/4	Divisional branch to 3rd toe tethered	Neuroma seen	Yes, but difficult	Good
4b		L 3/4	Nerve passing abnormally behind 3rd metatarsal distally	Neuroma seen	Not possible	Good
5	58 M	L 3/4	Nerve thickened	No neuroma Nerve thickened	Yes	Excellent
6	70 F	L 3/4	Thickening of the divisional branch to the 4th toe	No neuroma seen Nerve thickened	Yes	Fair
7	41 F	R 3/4	Nerve passing abnormally under 3rd MT neck distally. Bursal thickening	No neuroma seen Nerve thickened	Not possible	Poor
8	39 F	R 3/4	Neuroma interestingly on the divisional branch to the 3rd toe	Neuroma seen	Yes	Good
9	33 F	L 3/4	Neuroma tethered	Neuroma seen	Yes, difficult	Good
10	43 F	L 3/4	Difficult procedure due to overlying prominent vessels	Neuroma seen	Yes, but difficult	Good
11	59 F	R 3/4	Main nerve under 3rd MT shaft	No neuroma seen Nerve normal	Not possible	Good
12	60 M	L 3/4	Small divisional branch to 4th toe with a prominent plantar branch	Neuroma seen	Not possible	Excellent
13	42 F	L 3/4	Thickened nerve. Bursal thickening	No Neuroma seen Nerve thickened	Yes	Good
14	31 M	L 3/4	Neuroma interestingly being on the divisional branch to the 3rd toe	Neuroma seen Nerve thickened	Yes	Good
15	57 F	L 2/3	Nerve tethered with a prominent plantar branch. Bursal thickening	No neuroma seen Nerve thickened	Not possible	Poor
16	43 F	L 3/4	Neuroma mainly on the divisional branch to the 3rd toe with a prominent plantar branch	Neuroma seen	Yes, but after dividing the plantar branch	Excellent

In this study, the small plantar nerve branches arising proximal to the bifurcation of the interdigital nerve were severed to mobilise the nerve for dorsal transposition. In addition, in three patients, we observed a prominent plantar branch arising from the distal part of the common interdigital nerve proximal to the bifurcation.

### Complications

Two patients had superficial wound infections which were treated with a course of antibiotics with no sequelae. One patient had a DVT which was treated with 6 months of anticoagulation and had an excellent outcome. As DNT takes a longer surgical time, one can consider just dividing the DTIML in patients with high risk of DVT. Dividing only the DTIML results in rapid and stable improvement in 83% of patients [10].

## Discussion

Surgical management of Morton’s neuroma is frequently offered to patients who have failed non-operative management. Over the last decade, infiltrative and minimally invasive procedures have gained popularity.

Our results of DNT (84%) are marginally inferior when compared to the studies in literature which reported successful results in over 90% of the patients [4, 5, 11]. A previous comparative study with dorsal neurectomy showed that 92% successful results were obtained with dorsal transposition, but the number of patients was small [12].

### Multiple neuromas

One patient had three neuromas transposed, two adjacent neuromas in the same foot and the 3rd in the opposite foot with a good result. Two patients had bilateral neuroma transposed, with one having a good result on both sides, and the other had an excellent result on one side and a fair result on the opposite side. The occurrence of multiple Morton’s neuromas varies widely, from 4 to 65% [13–15]. Multiple studies reported lower patient satisfaction with excision of adjacent interdigital space neuromas which can lead to increased complications related to wound healing and numbness of the entire toe [16, 17].

In a systematic review, in 14 studies the nerve was excised, and in 4 studies it was decompressed by division of the deep transverse intermetatarsal ligament. There was an 88% success rate for neurectomy versus 94% success rate for the decompression; in the 2 studies on minimally invasive decompression surgery, success rate was 88% [18]. In a recent article on minimally invasive nerve decompression in 27 procedures in 25 patients, poor results were reported in 9 (40.9%) [19].

Satisfactory outcome of neurectomy ranges from 80 to 96% [7, 20]. In general, the recurrence of symptoms is accompanied by the formation of a stump neuroma with neural regeneration in 20% of the patients regardless of the approach [7, 21]. Intermuscular neuroma transposition after resection resulted in 100% successful results when compared with resection only with 50% decrease in pain in 86% of the patients [22, 23]. Despite these modifications, complete resection of the affected nerve remains the mainstay of surgical intervention for Morton’s neuroma.

### Poor results

Of the 6 patients with poor results, 3 had standard dorsal neuroma transposition and the other 3 had some anatomical variation (Table 4).

**Table 4:** Further details of the 6 patients with poor results

Patient	Age/sex	Level	Pre/post Giannini score	Follow-up duration	Intraoperative findings
1	61/F	L2/3	20/40	14 years	Prominent plexus of veins dorsal to the bifurcation but no neuroma seen, nerve dissected free and transposed
2	57/F	L2/3	20/40	8 years	Nerve tethered with large plantar branch and bursal thickening
3	41/F	R 3/4	10/40	12 years	Nerve passing abnormally under 3rd MT neck distally with bursal thickening

In the first patient, it was not clear what the exact cause of persistent pain (possible misdiagnosis, as intraoperatively notes suggested that there were abundant congested blood vessels in the web space which could possibly be causing nerve irritation).

In the second patient, the nerve was tethered with a large plantar branch and bursal thickening, and in the 3rd patient the nerve had an abnormal course under the 3rd metatarsal and bursal thickening**.** The other 3 patients had undergone standard dorsal neuroma transposition. However, no patient underwent a further surgical procedure. Of these 3 patients who underwent standard DNT procedure with poor results, one patient had further laser treatment, with improvement but still experiencing pain; 2 patients had a steroid injection with some benefit. These patients were offered further surgery (dorsal neurectomy) but they declined. The other 3 patients with anatomical variations (Table 4) were not keen on further surgery, and preferred to be left alone.

In a cadaveric study of five fresh-frozen feet, the common digital nerve demonstrated consistently small plantarly directed nerve branches up to 4 cm proximal to the transverse inter metatarsal ligament [11]. These small plantar branches can prevent proximal retraction of the transected nerve stump causing high incidence of recurrence of pain or a traumatic neuroma formation in the divided branches [24].

Villas et al. [25] reported recurrence of symptoms in one of the 23 patients in whom the nerve was preserved; the patient did well following resection of the nerve. Bradley et al. [26] reported unsatisfactory results in 4 of the 5 patients re-explored (80%), whereas Mann and Reynolds [7] reported significant improvement in 9 of 11 patients (81%). The plantar approach has been recommended for re-exploration for a recurrent neuroma [6, 27]. Patients should be carefully told that results are less than complete satisfaction in 20–40% of cases regardless of the incision used for the re-explorations [28].

Although 16% of patients reported poor results, none developed disastrous complications such as stump neuroma or complex regional pain syndrome.

Given the anatomical variants of the interdigital nerve and the multifactorial aetiology of Morton’s neuroma, nerve preserving procedures should be considered as primary surgical treatment rather than neurectomy. Dividing the DTIML/dorsal neurolysis should be the primary surgical treatment. Dorsal neurectomy can be offered to patients with poor results following resection of DTIML or Dorsal neurolysis. DNT is an option after dorsal neurolysis, but is technically demanding, takes longer and in some patients may not be possible because of anatomical variations.

We are aware of the limitations of the present study. For example, all the procedures were performed by a single surgeon. Despite sacrificing the small plantar branches for dorsal transposition, there is a risk of potential complications of complex regional pain syndrome or stump neuroma, but none were observed in this study. Anatomical variations in the interdigital nerve make it difficult or unable to transpose the nerve dorsally but this is unlikely to affect outcomes. Our study, on the other hand, presents several strengths. We used validated patient outcome measures, and long-term results of DNT are only slightly worse to those of dorsal neurectomy.

## Conclusion

Our overall results were marginally inferior when compared to what reported in the literature. Despite noticing anatomical variations in 40% of web spaces either of the nerve or in the web space, we could not identify any specific prognostic or risk factors in relation to patient outcomes. In this study, 19% of patients regretted having DNT surgery. Considering these factors, i.e. anatomical variations, increased operative time and failure to transpose the nerve, dividing the DTIML or dorsal neurolysis should be considered as the primary surgical treatment, and if this fails then neurectomy would be a secondary surgical procedure. Dorsal nerve transposition can be considered as a secondary surgical procedure if one is concerned about stump neuroma, but this may be difficult to perform due to anatomical variations and, in some patients, it may not be possible. Also, just dividing the DTIML can be considered in patients who are high risk of DVT as this is a quicker procedure.

## Data Availability

The data that support the findings of this study are available from [3rd party name] but restrictions apply to the availability of these data, which were used under license for the current study, and so are not publicly available. Data are, however, available from the authors upon reasonable request and with permission of [3rd party name].

## References

[1] Wu KK (1996). Morton’s interdigital neuroma: a clinical review of its etiology, treatment, and results. J Foot Ankle Surg.

[2] Gilmore WN (1973). Morton's metatarsalgia [abstract]. J Bone Joint Surg.

[3] Dellon AL (1992). Treatment of Morton's neuroma as a nerve compression. The role for neurolysis. J Am Podiatr Med Assoc.

[4] Diebold PF, Daum B, Dang-Vu V, Litchinko M (1996). True epineural neurolysis in Morton's neuroma: a 5-year follow up. Orthopaedics.

[5] Vito GR, Talarico LM (2003). A modified technique for Morton's neuroma. Decompression with relocation. J Am Podiatr Med Assoc.

[6] Johnson JE, Johnson KA, Unni KK (1998). Persistent pain after excision of an interdigital neuroma. Results of reoperation. J Bone Joint Surg (Am).

[7] Mann RA, Reynolds JC (1983). Interdigital neuroma: a critical clinical analysis. J Foot Ankle Surg.

[8] Giannini S, Bacchini P, Ceccarelli F, Vannini F (2004). Interdigital neuroma: clinical examination and histopathologic results in 63 cases treated with excision. Foot Ankle Int.

[9] Coughlin MJ (1991). Treatment of bunionette deformity with longitudinal diaphyseal osteotomy with distal soft tissue repair. Foot Ankle.

[10] Gauthier G (1979). Thomas Morton's disease: a nerve entrapment syndrome. A new surgical technique. Clin Orthop Relat Res.

[11] Okafor B, Shergill G, Angel J (1997). Treatment of Morton’s neuroma by neurolysis. Foot Ankle Int.

[12] Koti M, Sharma H, Parikh M, Edwards M, McAllister J (2002). Comparative analysis of dorsal nerve relocation versus dorsal neurectomy in surgical management of Morton’s neuroma. J Foot Ankle Surg.

[13] Mahadevan D, Venkatesan M, Bhatt R, Bhatia M (2015). Diagnostic accuracy of clinical tests for Morton’s neuroma compared with ultrasonography. J Foot Ankle Surg.

[14] Thompson FM, Deland JT (1993). Occurrence of two interdigital neuromas in one foot. Foot Ankle.

[15] Valero J, Gallart J, González D, Deus J, Lahoz M (2015). Multiple interdigital neuromas: a retrospective study of 279 feet with 462 neuromas. J Foot Ankle Surg.

[16] Kasparek M, Schneider W (2013). Surgical treatment of Morton's neuroma: clinical results after open excision. Int Orthop.

[17] Benedetti RS, Baxter DE, Davis PF (1996). Clinical results of simultaneous adjacent interdigital neurectomy in the foot. Foot Ankle Int.

[18] Valisena S, Petri GJ, Ferrero A (2018). Treatment of Morton’s neuroma: a systematic review. J Foot Ankle Surg.

[19] Archuleta AF, Darbinian J, West T, Ritterman Weintraub ML, Pollard JD (2020). Minimally invasive intermetatarsal nerve decompression for Morton's neuroma: a review of 27 cases. J Foot Ankle Surg.

[20] Bennett GL, Graham CE, Mauldin DM (1995). Morton's interdigital neuroma: a comprehensive treatment protocol. Foot Ankle Int.

[21] Barret SL, Pignetti TT (1994). Endoscopic decompression for intermetatarsal nerve entrapment—the EDIN technique: preliminary study with cadaveric specimens; early clinical results. J Foot Ankle Surg.

[22] Colgrove RC, Huang EY, Barth AH, Greene MA (2000). Interdigital neuroma: intermuscular neuroma transposition compared with resection. Foot Ankle Int.

[23] Thomson CE, Gibson JN, Martin D (2004). Interventions for the treatment of Morton’s neuroma. Cochrane Database Syst Rev.

[24] Amis J, Siverhus SW, Liwnicz BH (1992). An anatomic basis for recurrence after Morton's neuroma excision. Foot Ankle.

[25] Villas C, Florez B, Alfonso M (2008). Neurectomy versus neurolysis for Morton's neuroma. Foot Ankle Int.

[26] Bradley N, Miller WA, Evans JP (1976). Plantar neuroma: analysis of results following surgical excision in 145 patients. South Med J.

[27] Young G, Lindsey J (1993). Etiology of symptomatic recurrent interdigital neuromas. J Am Podiatr Med Assoc.

[28] Mann RA, Coughlin MJ, Mann RA (1999). Diseases of the nerve. Surgery of the foot and ankle.

